# X-linked ichthyosis associated with psychosis and behavioral abnormalities: a case report

**DOI:** 10.1186/s13256-017-1420-2

**Published:** 2017-09-22

**Authors:** Amna Malik, Ahmed Bait Amer, Mohammed Salama, Bander Haddad, Muhammad T. Alrifai, Mohammed Al Balwi, William Davies, Wafaa Eyaid

**Affiliations:** 1 King Abdullah International Medical Research Center (KAIMRC), Riyadh, Saudi Arabia; 2 King Saud Bin Abdulaziz University for Health Science, Riyadh, Saudi Arabia; 3 King AbdulAziz Medical City, Riyadh, Saudi Arabia; 4 Medical Research Council Centre for Neuropsychiatric Genetics and Genomics and Division of Psychological Medicine and Clinical Neurosciences, Cardiff University, Cardiff, UK; 5 School of Psychology, Cardiff University, Cardiff, UK; 6 Neuroscience and Mental Health Research Institute, Cardiff University, Cardiff, UK; 7 Department of Pediatrics MC 1510, King AbdulAziz Medical City, King Fahad National Guard Hospital, P.O Box 22490, Riyadh, 11426 Saudi Arabia; 8 MRC Centre for Neuropsychiatric Genetics and Genomics, Hadyn Ellis Building, Maindy Road, Cardiff, CF24 4HQ UK

**Keywords:** Attention deficit hyperactivity disorder, Autism spectrum disorder, Case report, Epilepsy, Psychosis, Steroid sulfatase, X-linked ichthyosis

## Abstract

**Background:**

X-linked ichthyosis is a dermatological condition caused by deficiency for the enzyme steroid sulfatase. Previously, X-linked ichthyosis/steroid sulfatase deficiency has been associated with developmental and neurological phenotypes. Here, we show for the first time, that X-linked ichthyosis may be comorbid with an additional psychiatric phenotype (psychosis).

**Case presentation:**

We report the case of an 11-year-old Saudi Arabian boy with X-linked ichthyosis associated with psychosis, mental retardation, autism spectrum disorder, inattentive attention deficit hyperactivity disorder, and epilepsy. Genetic analysis revealed a 1.68 Mb deletion encompassing *STS* in 95% of cells while biochemical analysis revealed correspondingly low steroid sulfatase activity consistent with a diagnosis of X-linked ichthyosis. The psychotic symptoms could be reasonably well controlled by administration of an atypical antipsychotic.

**Conclusions:**

This report describes a case of comorbid X-linked ichthyosis and psychosis (most closely corresponding to early-onset schizophrenia) for the first time, and suggests that deficiency for steroid sulfatase and contiguous genes may increase vulnerability to psychosis as well as other psychological disorders.

## Background

The ichthyoses are a group of dermatological conditions characterized by dry scaly skin; most of these disorders are hereditary, but examples of sporadic or acquired forms have been reported. Inherited ichthyoses are usually apparent during the first year of life, often at birth, and continue to affect a person throughout life [1]. In 1965, Wells and Kerr first recognized X-linked ichthyosis (XLI) in 81 affected males [2]. Approximately 90% of cases of XLI are caused by deletions within, or encompassing, the steroid sulfatase (*STS*) gene on chromosome Xp22.31, with ~ 10% caused by point mutations within the *STS* gene [3]; deletions may be “typical” (encompassing *STS* and a small number of adjacent genes), or “atypical” (encompassing many contiguous genes). XLI may occur solely as a skin disorder or may be associated with other physical findings such as corneal opacities (up to 50% of cases), cryptorchidism (20% of cases), chondrodysplasia punctata, and nephrotic syndrome [4–6]. Deletions encompassing *STS* have been reported to be associated with multiple behavioral, cognitive, and neurological phenotypes notably: mental retardation, developmental conditions including autism spectrum disorders (ASDs), attention deficit hyperactivity disorder (ADHD), and seizures [7]. In the first systematic behavioral study of boys with XLI, Kent *et al*. showed high rates of ADHD (particularly inattentive ADHD) and autism-related conditions; the latter were seen when the deletions were large and included the neuroligin 4 gene (*NLGN4X*) [8]. Consistent with these data, genetic variants within *STS* are associated with inattentive symptoms in children with ADHD [9, 10] while mice lacking the *Sts* gene exhibit behavioral phenotypes associated with developmental disorders including inattention, hyperactivity, elevated emotional reactivity, behavioral perseveration, and aggression [11–13]. *STS* is expressed in brain regions associated with attention, impulsivity, and the integration of, and response to, sensory information [10]. XLI rarely interferes with the activities of daily living and individuals with mild forms may not require treatment [4]. The skin condition can be managed relatively effectively, but some patients may experience a significantly reduced quality of life due to self-consciousness and social embarrassment or due to comorbid behavioral, cognitive, or neurological conditions; such patients may require lifelong treatment [14, 15].

## Case presentation

A summary of the case presentation and management is provided in Table 1. An 11-year-old Saudi Arabian boy, in Grade Three of primary school, initially presented with a year-long history of epileptic fits in the form of abnormal jerky movement of upper limbs accompanied by fixed eye gaze. The episodes typically lasted for a few seconds each, with up to 15 episodes per day, and were often preceded by episodes of unexplained crying.

**Table 1:** Timeline of case presentation

Timeline	Symptoms and treatment
Birth	Born at 28 weeks’ gestation: low birth weight with undefined congenital ichthyosis
Early childhood	Language and gross motor milestones delayed; poor social interaction and restricted play; learning disability and poor academic performance
Initial presentation at 11 years	Year-long history of epileptic fits and coincident behavioral abnormalities; initial treatment with herbal remedies before consultation with physicians at local hospital. Presented to local hospital with acute symptoms of agitation and unresponsiveness; began to believe that others were watching him. Was seen talking to himself and laughing without reason. Electroencephalograph abnormalities detected and carbamazepine and olanzapine treatment initiated, but ineffective due to poor patient adherence
One month later	Referred as emergency admission to tertiary hospital with acute psychotic episode (with decline in daily functioning and double incontinence); microcephaly and ichthyosis noted. Three-week treatment with lorazepam initiated
Three weeks later	Referral to dermatology, genetics, neurology, and child psychiatry clinics. Genetic and biochemical confirmation of steroid sulfatase deficiency (X-linked ichthyosis), and detection of autosomal regions of homozygosity. Normal gross brain structure reported with electroencephalograph abnormalities. Methylprednisolone pulse therapy and immunoglobulin regime initiated to treat suspected encephalitis, but ineffective in treating cognitive symptoms after 5 days. Olanzapine treatment initiated which brought psychiatric symptoms under control after 2 weeks
Follow-up	No psychotic symptoms evident; patient maintained on olanzapine for 1 year following hospital discharge. Diagnoses of autism spectrum disorder and attention deficit hyperactivity disorder made

The boy was born at 28 weeks’ gestation by spontaneous vaginal delivery after an uneventful and uncomplicated pregnancy. He had a low birth weight of 1.5 kg (below the 5th centile). During pregnancy, his mother took no medication apart from folic acid supplementation during the first trimester. Following delivery, he was kept in the intermediate care unit for 3 months until he gained sufficient weight. At birth, he was diagnosed with congenital ichthyosis although the subtype was not defined; from early childhood, his parents were advised by the dermatologist of the importance of daily bathing and regular application of emollient creams containing urea. His language and gross motor milestones were delayed; he had poor social interaction with others and restricted play. There was a family history of developmental disorders (speech and language problems and learning disability), and the boy’s 13-year-old sister exhibited mild mental retardation. There was no family history of ichthyosis, or psychotic or mood disorder.

The boy’s parents reported that his academic performance was poor, that he had a learning disability and that he required substantial support in school. His parents also reported that, coincidentally with the epileptic fits, he started exhibiting behavioral changes, becoming socially withdrawn, unresponsive, and preoccupied. He also became restless and wandered aimlessly, shouted and made odd noises, began to believe that others were watching him, and was seen talking to himself and laughing without any reason. His parents initially sought help from traditional healers who provided him with herbal medicine for a short period of time (honey and herbal oil). After no improvement in the boy’s symptoms, his parents arranged for him to be seen by a local psychiatrist and a neurologist. According to the boy’s father, initial blood test results (which included full blood counts, liver function test, urea and electrolytes, and thyroid function test) were normal; however, an electroencephalograph (EEG) showed epileptiform discharges. Carbamazepine (100 mg per day) and olanzapine treatment (5 mg, twice per day) were instigated to treat the boy’s EEG abnormalities and his disturbed behavior respectively; carbamazepine was considered to be the optimum treatment to alleviate his brief episodes of staring with unresponsiveness which were suggestive of complex partial seizures. However, his adherence to the treatment regime was poor: he did not take his medication as prescribed and had no regular follow-up. Hence there was minimal improvement in his condition.

One month after his initial presentation to local doctors, the boy was urgently referred and admitted to a tertiary hospital. He was extremely aggressive and attempted to bite his parents and siblings; he was also verbally abusive, highly vocal (screaming), and restless. His sleep pattern was extremely disturbed; he remained awake for the majority of the 3 days prior to admission. He became doubly incontinent. On examination, the admitting doctor documented microcephaly and dry skin with scales over his body (which the patient was peeling off and eating); there was no erythema. His vital signs were stable, and there were no neurological signs apart from eye twitches. He was shouting and uncooperative, and was looking around as if responding to visual stimuli. He was also talking to himself and mumbling. He was disoriented with regard to time and place, and demonstrated poor general knowledge, even in a simple color-naming task. He was restless and aggressive, required sedation, and lorazepam (0.5 mg twice per day) was administered orally from the day of admission. He showed immediate improvements in his sleep pattern and became less agitated and restless. However, he persisted in talking to himself and continued to be suspicious and paranoid. His presentation and symptoms were considered to best resemble the *Diagnostic and Statistical Manual of Mental Disorders*, Fifth Edition (DSM-5.0) diagnostic criteria for early-onset schizophrenia [16]. Therefore, he continued to be treated with lorazepam for a 2-week period, with treatment tapering off within the third week.

He was subsequently referred to several subspecialties including dermatology, genetics, neurology, and child psychiatry. During the hospital course, a number of physiological and metabolic measures were taken (Table 2).

**Table 2:** Levels of serum and cerebrospinal fluid metabolites in our patient relative to normative values

Measures	Patient’s values	Normal range
Serum		
WBC	6.1 × 10^9^	4.0–12.0 × 10^9^/L
Hemoglobin	12.9	113–150 g/L
Urea	4	2.5–6 mmol/L
Sodium	137	138–145 mmol/L
Potassium	4	3.4–4.7 mmol/L
Chloride	103	95–110 mmol/L
CO_2_	22	20–28 mmol/L
Creatinine	46	27–62 mmol/L
Albumin	39	38–54 g/L
Calcium	2.2	2.2–2.7 mmol/L
Phosphorus	1.7	1.05–1.85 mmol/L
Magnesium	0.81	0.7–0.85 mmol/L
Total bilirubin	18	3.4–20.5 umol/L
AST	26	5–34 U/L
ALT	15	5–55 U/L
TP	66	60–80 g/L
ESR	20	0–15 mm/hour
C-reactive protein	<3.5	<3.5
GTP	45	12–64 U/L
Ammonia	61	18–72 umol/L
TSH	3.01	0.35–4.94 mIU/L
FT4	10.7	9–19 pmol/L
TSH antibodies	Negative	Negative
FT4 antibodies	Negative	Negative
TSH hormone receptor antibodies	0.5 (Negative)	<1.8 IU/L
CK	128	30–200 U/L
Copper	22.9	11.78–22.77 umol/L
Pyruvate	0.315	0.08–0.16 mmol/L
Vitamin D	146.9	62.6–228.0 pmol/L
Vitamin A	687	300–700 ug/L
Ceruloplasmin	0.36	0.20–0.60 g/L
Carbamazepine level	27	17–42 umol/L
Homocysteine level	10	≤15.0 umol/L
Lead level	<0.18	≤1.22 umol/L
Mycoplasma IgM	3.6	<10 index is negative
Mycoplasma IgG	<10.0	<10.0 AU/ml negative
EBV	<10	<20 U/ML negative
Cytomegalovirus	<5.0	<12.0 U/ml negative
Antistreptolysin O titer	286	>200 IU
*Brucella* antibodies	<1:160	<1:160 is negative
C3	1.45	0.80–1.70 g/L
C4	0.09	0.14–0.44 g/L
Antinuclear antibodies	0.50	<1.5 index is negative
Anti-DNA	17	<200 IU/ml negative
Anti-NMDA antibodies	Negative	Negative
Glutamate receptor IgG antibodies	Not detectable	Not detectable
Very long chain fatty acids	Unremarkable	-
Biotinidase	5.80	4.20–12.80 nM/minute/ml
Lactic acid	1.54	0.50–2.20 mmol/L
Newborn screening	Unremarkable	-
Cerebrospinal fluid		
Glucose	3.7	3.30–4.40 mmol/L
Protein	0.44	0.15–0.40 g/L
RBC	11	Not significant
WBC	<1	Not significant
Lactic acid	1.2	≤2.20
*Brucella* (IgM, IgG)	<10	Unremarkable
Measles (IgM, IgG)	Negative	-
Cytomegalovirus (PCR)	Negative	-
HSV (PCR)	Not detected	-
Anti-NMDA antibodies	Negative	-
Mycoplasma antibody	Negative	-
Culture and sensitivity	Negative	-

*ALT* alanine aminotransferase, *AST* aspartate aminotransferase, *CK* creatine kinase, CO_2_ carbon dioxide, *C3* complement component 3, *C4* complement component 4, *DNA* deoxyribonucleic acid, *EBV* Epstein–Barr virus, *ESR* erythrocyte sedimentation rate, *FT4* free thyroxine, *GTP* glutamyl transpeptidase, *HSV* herpes simplex virus, *IgG* immunoglobulin G, *IgM* immunoglobulin M, *NMDA* N-methyl-D-aspartate, *PCR* polymerase chain reaction, *RBC* red blood cells, *TP* total protein, TSH thyroid-stimulating hormone, *WBC* white blood cells

Most of our patient’s results were within the normal range, although relatively low levels of hemoglobin and relatively high values for erythrocyte sedimentation rate (ESR) and pyruvate were noted.

On the basis of family history and skin appearance, dermatologists suspected XLI and continued prescribing emollient creams containing urea. Chromosomal G-banding and fluorescence *in situ* hybridization (FISH) analysis on peripheral blood and fibroblast cultures revealed an interstitial deletion within Xp22.31 on 95% of the examined cells with a low level of mosaicism (5% of cells with normal karyotype). Consistent with the FISH analysis, STS activity in fibroblasts was low at 3.5 nmol/hour/protein (normal range, 5 to 32 nmol/hour/protein). The boy’s genomic deoxyribonucleic acid was assayed on a combined comparative genomic hybridization (CGH) and single nucleotide polymorphism (SNP) array in an attempt to specify a genetic basis for his clinical presentation. The array CGH study confirmed a 1.68 Mb deletion within the cytogenetic band Xp22.31 (hg 19: 6,456,036-8,139,238) that contains the *STS* gene in addition to *VCX3A*, *PUDP*, *VCX,* and *PNPLA4* genes, and two microRNAs (*MIR4767* and *MIR651*) [17]. His mother was tested for mutation carrier status, but his siblings were not genetically tested. Eight regions of homozygosity spanning ~ 111Mb in total were detected within our patient across multiple autosomes (Table 3). Subsequent targeted exome sequencing of these homozygosity blocks using next generation sequencing and capillary Sanger sequencing did not reveal any pathological, likely pathological, or other sequence change of possible clinical relevance in the coding regions of the genes within these regions.

**Table 3:** Regions of runs of homozygosity within our patient based on human genome build GRCh37/hg19

Chromosome	Cytogenetic band	Genomic location	Size of run (bp)
6	p25.3-p25.1	211,941-5,861,181	5,649,241
6	p24.3-p21.2	10,297,504-37,700,562	27,403,059
6	q12-q14.1	69,878,396-78,700,562	8,266,676
6	q21-q22.33	110,749,132 -128,732,846	17,893,715
7	p13-p12.1	44,162,384-52,104,941	7,942,558
10	q23.31-q25.2	91,297,540-113,415,612	22,118,073
11	p12-p11.12	36,892,152-49,401,125	12,508,974
11	q11-q13.1	55,196,818-64,306,768	9,109,951
		Total ROH size:	110,982,247

*bp* base pairs, *ROH* runs of homozygosity

Radiological and neurological investigations using computed tomography (CT) revealed a structurally normal brain. A CT venogram showed no evidence of cerebral venous thrombosis. Magnetic resonance imaging (MRI) indicated mild periventricular leukomalacia, possibly related to our patient’s premature birth. Two EEGs were performed; the first one performed initially during his stay in the hospital and the second one was 2 months after his discharge. The first EEG showed mild slowing of the background with no evidence of any epileptiform discharges; the second EEG showed mild, but improved, slowing with no evidence of epileptiform discharge. An autoimmune etiology was entertained by the neurologist; this was partially on the basis of a clear cerebrospinal fluid (CSF) and acute behavioral symptoms; thus, a methylprednisolone pulse therapy regime (50 mg/kg) to treat suspected encephalitis was initiated for a period of 5 days, in addition to intravenously administered immunoglobulin over another 5 days. These therapies did not improve our patient’s symptoms of disorientation, cognitive dysfunction, and psychosis. He showed some improvement in symptoms with a subsequent management regime of 5 mg olanzapine twice per day, but he demonstrated poor compliance with this. The psychiatry team opted to continue him on this drug but at higher doses (the dose was increased from 10 mg to 20 mg per day gradually over a 2-week period) to control his psychotic symptoms, as per prescribing guidelines [18]; olanzapine is a second-generation antipsychotic which has been shown to have robust clinical benefits for treating early-onset schizophrenia [19]. With this revised treatment regime, his psychiatric symptoms were brought under control, and eventually he was discharged with follow-up appointments in ophthalmology (for bilateral iris mammillation), psychiatric, pediatric neurology, and dermatology clinics.

The follow-up by the psychiatric clinic revealed no psychotic symptoms as indexed by the mental state examination; he was maintained on olanzapine 15 mg per day for almost 1 year after he was discharged from our hospital. Neuropsychological testing revealed a non-verbal intelligence quotient (IQ) of 57 with Test of Nonverbal Intelligence, Second Edition (TONI-2), consistent with a mild intellectual disability, and an ASD on the basis of the Autism Diagnostic Observation Schedule (ADOS). A diagnosis of ADHD (inattentive subtype) was also made on the basis of parental history, academic reports, and Conners Questionnaire; psychostimulant medication was not considered to have been beneficial in treating his ADHD symptoms.

## Discussion

Our patient presented with dry scaly skin and pronounced behavioral and neurological symptoms that included: psychotic features, disorganized behavior, ADHD (inattentive subtype), ASD, and a learning disability. His EEG showed epileptiform discharges. Pharmacological treatments to control the psychotic symptoms and epilepsy were relatively effective. Genetic and biochemical analysis confirmed the diagnosis of XLI.

It is feasible that the mutation encompassing the *STS* gene and some, if not all, of the behavioral and neurological features seen in our patient are causally related. The limited clinical literature documents several cases of children in which XLI is comorbid with epilepsy/EEG abnormalities, mental retardation, and ADHD or an autism-related condition (particularly in the case of large chromosomal deletions) [8, 20, 21], while animal studies have emphasized a link between *Sts* deficiency and cognitive and behavioral phenotypes associated with developmental disorders [11–13].

To the best of our knowledge, this is the first report of psychosis consistent with a diagnosis of early-onset schizophrenia in a child with confirmed XLI. Given the comparative rarity of both XLI and psychosis in the general population, our observations, together with previous literature reporting paranoid schizophrenia in two females with deletions spanning *STS* [22] and implicating STS deficiency in postpartum psychosis [23, 24], suggest that, in addition to influencing risk of developmental disorders, STS deficiency may also predispose to risk of psychotic illness; this notion is consistent with the latest genome-wide genetic screens which show that individual polymorphisms or mutations may act as risk factors in multiple developmental and affective conditions [25].

Our patient’s behavioral and neurological presentation could feasibly be modulated by other genetic or environmental factors segregating within his family; this idea is supported by the strong family history of learning disability and the communication deficits in our patient’s eldest sister. In addition, autosomal runs of homozygosity could theoretically have contributed toward our patient’s clinical presentation through revealing recessive deleterious mutations of relevance to brain function [26]; however, the fact that exome sequencing detected no coding mutations of clinical significance within these regions makes this explanation unlikely. Finally, it is possible that living with a congenital skin condition and/or treatment for that condition, for example retinoids or poorly specified traditional and herbal remedies, may influence psychological function. There is a case report of a woman with ichthyosis vulgaris who presented with a schizophrenia-like psychosis and EEG abnormalities similar to our patient [27]; however, she also had a strong family history of psychosis and learning disability.

## Conclusions

This case and the previous limited literature highlight the severe behavioral, psychiatric, and neurological symptoms that may be comorbid with XLI. Clearly, given the prevalence of the condition (~1 in 3000 to 6000 men, equivalent to over 1.2 million individuals worldwide [4]), there is a need for the physical and psychological phenotypes associated with it (including the prevalence of psychosis) to be comprehensively described. Such an endeavor will allow individuals such as our case to have their condition and potential comorbidities recognized and treated at an early stage, and to be monitored closely by appropriate multidisciplinary teams.

## Abbreviations

ADHD: Attention deficit hyperactivity disorder
ADOS: Autism diagnostic observation schedule
ASDs: Autism spectrum disorders
CGH: Comparative genomic hybridization
CSF: Cerebrospinal fluid
CT: Computed tomography
DSM-5.0: *Diagnostic and statistical manual of mental disorders*, fifth Edition
EEG: Electroencephalograph
FISH: Fluorescence *in situ* hybridization
IQ: Intelligence quotient
MRI: Magnetic resonance imaging
*NLGN4X*: Neuroligin 4 gene
SNP: Single nucleotide polymorphism
STS: Steroid sulfatase
TONI-2: Test of nonverbal intelligence, second edition
XLI: X-linked ichthyosis
